# Actinomycotic Osteomyelitis of Maxilla Presenting as Oroantral Fistula: A Rare Case Report

**DOI:** 10.1155/2015/689240

**Published:** 2015-09-15

**Authors:** Ashalata Gannepalli, Bhargavi Krishna Ayinampudi, Pacha Venkat Baghirath, G. Venkateshwara Reddy

**Affiliations:** ^1^Department of Oral Maxillofacial Pathology and Microbiology, Panineeya Mahavidyalaya Institute of Dental Sciences & Research Centre, Kamala Nagar, Dilsukhnagar, Hyderabad, Telangana 500 060, India; ^2^Department of Oral Maxillofacial Surgery, Panineeya Mahavidyalaya Institute of Dental Sciences & Research Centre, Kamala Nagar, Dilsukhnagar, Hyderabad, Telangana 500 060, India

## Abstract

Actinomycosis is a chronic granulomatous infection caused by *Actinomyces* species which may involve only soft tissue or bone or the two together. Actinomycotic osteomyelitis of maxilla is relatively rare when compared to mandible. These are normal commensals and become pathogens when they gain entry into tissue layers and bone where they establish and maintain an anaerobic environment with extensive sclerosis and fibrosis. This infection spreads contiguously, frequently ignoring tissue planes and surrounding tissues or organ. The portal of entry may be pulpal, periodontal infection, and so forth which may lead to involvement of adjacent structures as pharynx, larynx, tonsils, and paranasal sinuses and has the propensity to damage extensively. Diagnosis is often delayed and is usually based on histopathology as they are cultured in fewer cases. The chronic clinical course without regional lymphadenopathy may be essential in diagnosis. The management of actinomycotic osteomyelitis is surgical debridement of necrotic tissue combined with antibiotics for 3–6 months. The primary actinomycosis arising within the maxilla with contiguous involvement of paranasal sinus with formation of oroantral fistula is rare. Hence, we present a 50-year-old female patient with chronic sclerosing osteomyelitis of maxilla which presented as oroantral fistula with suppurative and sclerotic features.

## 1. Introduction

Actinomycosis is a rare saprophytic infection that is characterized by granulomatous and suppurative lesions caused by resident oral microbiota, Actinomycetaceae.* Actinomyces* are filamentous bacteria which resemble fungi. They are slow growing Gram-positive, non-acid fast, anaerobic or microaerophilic bacteria. Most of the species isolated from actinomycotic lesions have been identified as* A. israelii*,* A. viscosus*,* A. odontolyticus*,* A. naeslundii*, or* A. meyeri* [1, 2]. It can present in an acute, subacute, or chronic form. It may involve only soft tissue or bone (osteomyelitis) or the two together. This infection typically spreads contiguously, frequently ignoring tissue planes and surrounding tissues or organs, ultimately producing multiple sinus tracts [2, 3]. It primarily affects soft tissues, rarely affecting bone; osseous involvement occurs rarely. Incidence of* Actinomyces* infection in mandible is 53.6%, followed by cheek (16.4%), chin (13.3%), maxilla (5.7%), and TMJ (0.3%) [2].

Actinomycotic osteomyelitis of maxilla is relatively rare when compared to mandible, probably because of better circulation which provides increased oxygen supply [3, 4]. The primary actinomycosis arising within the maxilla with contiguous involvement of paranasal sinus with formation of oroantral fistula and palatal perforations is rare [1, 4–7]. It has myriad clinical presentations and is a potentially benign and completely curable disease but if not detected early, it has the propensity to damage extensively. This unusual case shows chronic actinomycotic osteomyelitis of maxilla with suppurative and sclerosing features which presented as oroantral fistula in a 50-year-old female patient.

## 2. Case Report

A 50-year-old female patient presented with a complaint of nonhealing of tooth socket and an opening in the region of right first and second maxillary premolars with continuous pus discharge for three months. She gave a previous history of painful swelling in the same region one and half year back with pus discharge, gradual loosening, and exfoliation of 14, 15 one year later. She was put on pain medication and antibiotics by local doctors in and around their village, for which she has no records. There was no relief from medication, the swelling persisted, and she was referred to ENT hospital. There, she underwent surgery for drainage of abscess and was given one-week course of oral penicillin, 6 months back. There was no healing and the patient reported with nonhealing of opening in the maxilla and continuous pus discharge through the opening. Later, the patient was given a course of antifungal medication, that is, nystatin, but there was no improvement and she was referred to our hospital. Her general health was unremarkable with no known allergies.

**Figure 1:**
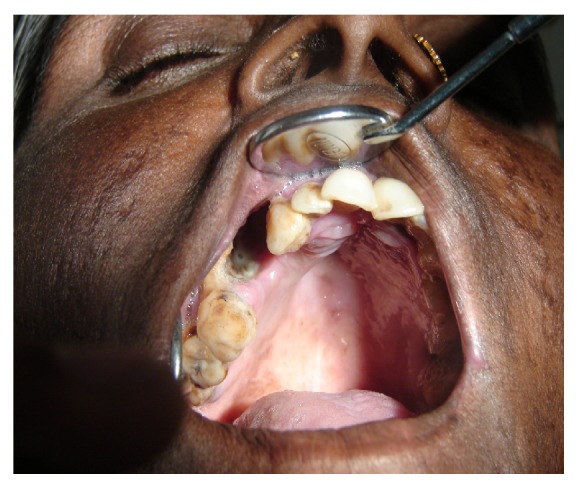
Clinical photograph showing oroantral fistula.

**Figure 2:**
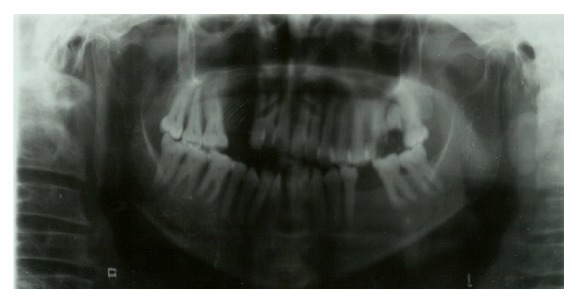
OPG showing destructive radiolucent lesion of right maxilla.

**Figure 3:**
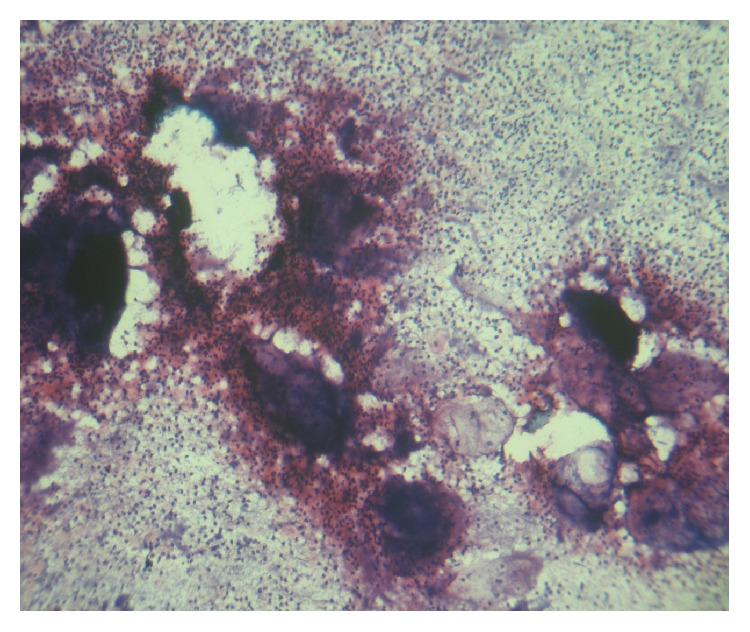
Photomicrograph of smear (10x) Gram's staining image of actinomycotic colony with peripheral neutrophilic aggregation.

**Figure 4:**
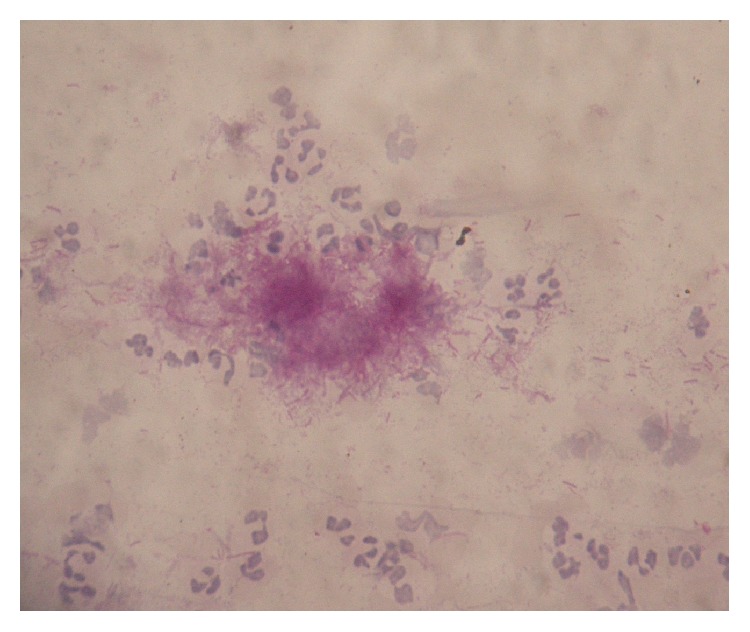
Photomicrograph of smear (oil immersion). Tangled mass of Periodic acid-Schiff (PAS) positive branching filaments.

An intraoral examination showed an irregular necrotic palatal defect with antral communication, forming an oroantral fistula in the region of 14, 15. There were necrosis and purulent discharge (Figure 1). There was no evidence of any lymphadenopathy. On palpation near the defect, it was nontender and the palate was soft to firm in consistency. Based on the history and clinical findings, provisional diagnosis of chronic inflammatory/infective lesion was given. The differential diagnosis of malignancies was considered. The patient was advised with Orthopantomogram (OPG), routine blood examination, incisional biopsy, and cytological smear, bacterial culture prepared from antral discharge. The OPG showed a destructive lesion involving palate (Figure 2). Routine hematological investigations were normal except for neutrophilic leucocytosis and elevated erythrocyte sedimentation rate (ESR). The antral discharge showed yellowish granules, which revealed actinomycotic colonies on smear preparation. There were clumps of tangled masses of elongated bacilli with peripheral neutrophilic aggregation (Figure 3). On Gram staining, it revealed Gram-positive intertwined branching filaments along with Gram-positive and negative cocci and bacilli. The filaments were non-acid fast and were PAS (Periodic acid-Schiff stain) positive (Figure 4).

**Figure 5:**
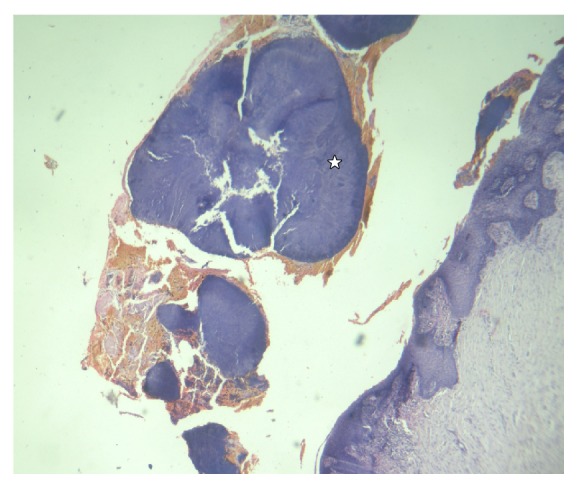
Photomicrograph of biopsy (10x) H&E oral epithelium with large actinomycotic colonies (☆).

**Figure 6:**
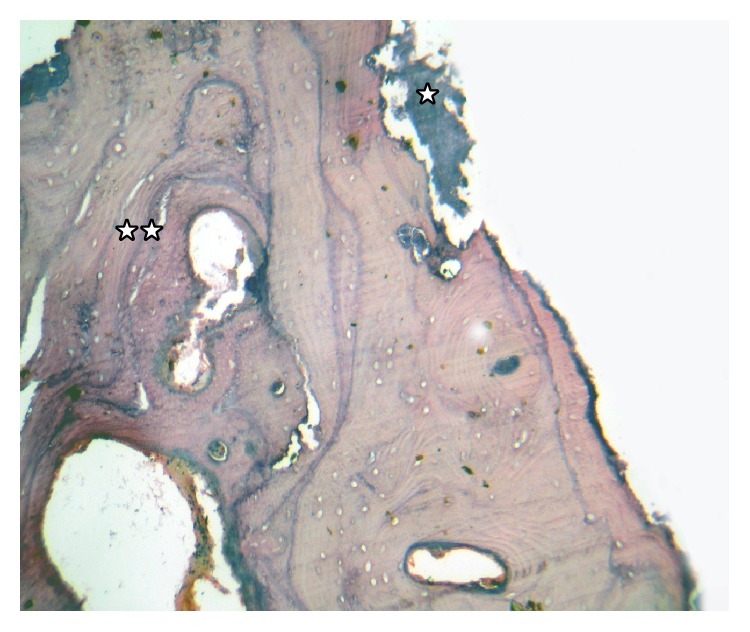
Photomicrograph (10x) of H&E decalcified section. Necrotic thickened bony trabeculae with* Actinomyces* filaments on trabecular surface of bony spicules (☆), with extensive sclerosis of bone showing prominent resting and reversal lines (☆☆).

**Figure 7:**
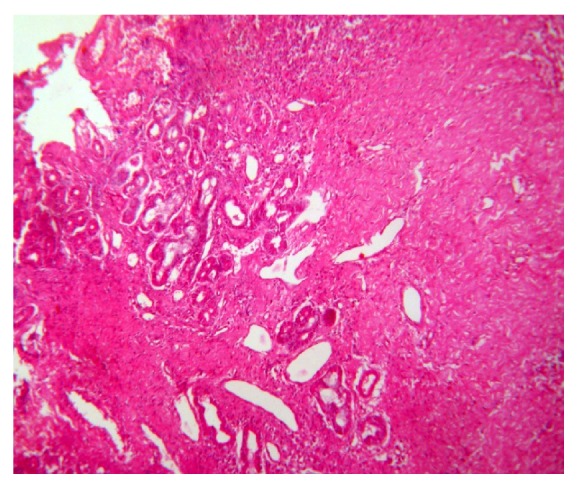
Photomicrograph (10x) H&E section. Extensive fibrosis with granulomas and degeneration of salivary gland acini.

**Figure 8:**
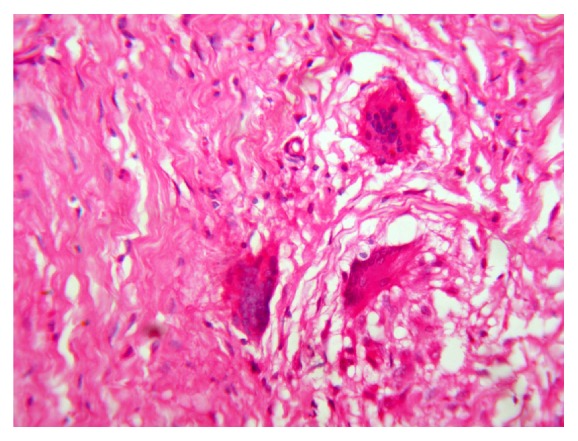
Photomicrograph (40x) of H&E. Resolving granulomas with giant cells.

**Figure 9:**
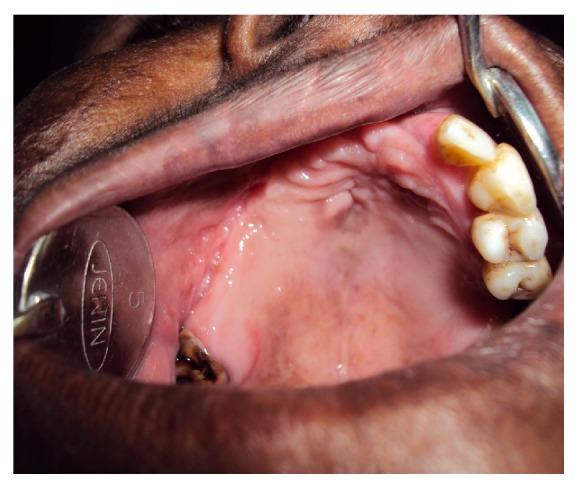
Postoperative photograph after 2 months of follow-up.

The incisional biopsy from the soft tissue and adjacent exposed necrotic bone revealed a stratified squamous epithelium with large clumps of basophilic bacterial colonies resembling actinomycotic colonies (Figure 5). The necrotic bone showed sclerosis with actinomycotic colonies within bony sequestra and on trabecular surface (Figure 6). Anaerobic bacteriological culture showed multiple varying sizes of colonies but could not isolate* Actinomyces* species. A diagnosis of actinomycotic osteomyelitis was reached. To evaluate deeper tissues and relieve pain, extraction of 21, 11, 12, 12, 16, and 17 and debridement of infected area, with removal of bony sequestra and buccal advancement flap to close the fistula was carried out and tissue was submitted for histopathological analysis. The patient was kept under penicillin IV one million units for 3 days and Augmentin 625 mg three times a day (tid) for one week and was reviewed after one week. There was dehiscence and wound opening in the same area. The patient was readmitted and extensive decortications with debridement of palate and antral wash were done. Surgical specimen was histologically evaluated which showed extensive sclerosis of bone with prominent resting and reversal lines. There was extensive fibrosis with resolving granulomas and no bacterial colonies were observed (Figures 7 and 8). She was treated with IV penicillin for 1 month and was kept on doxycycline 100 mg, twice a day (bid) for the following 2 months. The patient was recalled after one month and showed improvement with resolution of the lesion (Figure 9). The patient was followed up for six months with monthly recall and there was no recurrence.

## 3. Discussion

Actinomycosis in the maxilla accounts for only 0.5–9% of all head and neck cases [4, 5]. Although the pathomechanism of actinomycotic osteomyelitis is unclear, it is suggested that inflammation begins when the normal composition of the oral microbiota is disturbed, and chronic inflammation leads to localized pathological changes in the bone [5, 8]. The process of granulomatous replacement leads to foci of bony spicules rather than complete lytic destruction of bone. It causes sclerosing type of osteomyelitis mimicking bone tumors [2]. Marx et al. described a more refractory form of chronic diffuse sclerosing osteomyelitis produced by* Actinomyces *together with other fastidious organisms such as* Eikenella* and* Arachnia*. It has 2 : 1 predilection for women and an average course of nearly 5 years before diagnosis [9].

The primary osteomyelitis of maxilla with involvement of maxillary sinus could be a result of adjacent oral soft tissue involvement due to periapical or periodontal infection in this case. Oral infection may involve pharynx, larynx, salivary glands, tonsils, and paranasal sinuses [6, 7]. The prerequisite for the development of endogenous disease is the transport of pathogens into tissue layers with an anaerobic environment. The chronic purulent infection, poor oral hygiene with unhealed sockets, and surgical maneuvers in the present case show that the penetration site of these organisms into deeper tissues was from gingiva, periodontal disease, or chronic periapical abscess and oral surgical procedures facilitated pathogenicity of these organisms [5, 6]. These organisms have low potential for virulence and invasion but the companion bacteria act as copathogens and participate in the production of infection by elaborating a toxin or enzyme. This polymicrobial associate flora works in a synergistic fashion to form a specific ecosystem with low oxidoreduction potential favorable for anaerobic growth. It destroys highly vascularized aerobic system and replaces it with a poorly irrigated granulated tissue thereby permitting anaerobic milieu [2, 5]. Marx et al. described it as mutualism than synergism because sulfur granules are produced in tissues but not under laboratory conditions suggesting the importance and contribution of associate bacteria in colony formation, growth, and evasion of host defenses [9]. Infection presents itself as granulomatous inflammatory response with central suppurative necrosis consisting of aggregates of bacterial filaments surrounded by neutrophils. The outermost fibrotic envelope is collagenous, avascular permitting spread of bacteria. In the present case there was extensive suppuration with large actinomycotic colonies, granulomatous response, and extensive endosteal sclerosis with resting and reversal lines with colonies of organism within bony trabeculae. These features could be due to continuous exacerbation and regression of symptoms with on and off antibiotic course in the patient. The case radiographically presented as a destructive lesion and no evidence of bony sclerosis or periostitis was observed.

Experimental infections have shown that pure cultures of* Actinomyces* produce acute suppurative infections and mixed infections produce chronic persistent lesions with* Eikenella* and* A. actinomycetemcomitans*, which evade direct contact with antibiotics and leukocytes within sulfur granule, and are the basis for recommendation of high doses of antibiotics [2, 9].

The oral antibiotic course is usually ineffective as seen in the present case and should be used with surgical procedures. In chronic long standing cases, fibrosis can evolve into high level sclerosis; endosteal sclerosis can lead to therapeutic failure as poor vascularity inhibits penetration of antibiotics. The thickness of each sulfur granule colony, protective nature of sulfur granule, and the characteristic necrosis and fibrosis surrounding such infections require large diffusion gradients, making high doses a necessity [2, 3, 9]. Treatment should be vigorous with removal of granulation tissue and resection of sequestrated bone until healthy tissue is exposed. The drug of choice is penicillin with duration ranging from 3 to 12 months and erythromycin, tetracyclines are useful alternatives [2, 3, 8].

Diagnosis is often delayed because of varied presentations and they are cultured in fewer than 50% of cases and are the first element of diagnosis in fewer than 10% of cases [2]. In the present case the sulfur granules were observed in the antral discharge and confirmed by cytologic smear even though they could not be isolated by culture. The sulfur granules are not pathognomonic as they are produced by mycotic pathogens,* Nocardia*, Botryomycosis and since such instances are rare, Myerowitz states that the presence of sulfur granules virtually guarantees the diagnosis of actinomycosis [2]. A clinical diagnosis may be difficult and numerous problems are associated with bacteriological culture but histopathology is highly recommended. The nucleic acid probes and polymerase chain reaction (PCR) methods have been developed for rapid and accurate identification but are highly expensive [5, 10].

## 4. Conclusion

Oral mucous membrane is often the penetration site of* Actinomyces* into deeper tissues. Definitive diagnosis based on cultures is not often positive but histopathology is still the most reliable diagnostic aid. It is a potentially benign and completely curable disease with an early diagnosis and adequate therapy is absolutely essential so that there is minimal functional and esthetic damage.

## References

[1] Rubin M. M., Krost B. S. (1995). Actinomycosis presenting as a midline palatal defect. *Journal of Oral and Maxillofacial Surgery*.

[2] Miller M., Haddad A. J. (1998). Cervicofacial actinomycosis—review. *Oral Surgery, Oral Medicine, Oral Pathology, Oral Radiology, and Endodontics*.

[3] Marx R. E., Stern D. (2003). Inflammatory, Reactive, and infectious Diseases. *Oral and Maxillofacial Pathology—A Rational for Diagnosis and Treatment*.

[4] Crossman T., Herold J. (2009). Actinomycosis of the maxilla—a case report of a rare oral infection presenting in general dental practice. *British Dental Journal*.

[5] Sezer B., Akdeniz B. G., Günbay S., Hilmioğlu-Polat S., Başdemir G. (2013). Actinomycosis osteomyelitis of the jaws: report of four cases and a review of the literature. *Journal of Dental Sciences*.

[6] Damante J. H., Sant'Ana E., Soares C. T., Moreira C. R. (2006). Chronic sinusitis unresponsive to medical therapy: a case of maxillary sinus actinomycosis focusing on computed tomography findings. *Dentomaxillofacial Radiology*.

[7] Rastogi R., Rao D., Suma G. N., Bhargava S., Rastogi V., Rastogi K. (2011). Radiological features in actinomycosis of paranasal sinus region and base of skull with oro-antral fistula. *Journal International Medical Sciences Academy*.

[8] Bartkowski S. B., Zapala J., Heczko P., Szuta M. (1998). Actinomycotic osteomyelitis of the mandible: review of 15 cases. *Journal of Cranio-Maxillo-Facial Surgery*.

[9] Marx R. E., Carlson E. R., Smith B. R., Toraya N. (1994). Isolation of *Actinomyces* species and *Eikenella corrodens* from patients with chronic diffuse sclerosing osteomyelitis. *Journal of Oral and Maxillofacial Surgery*.

[10] Hansen T., Kunkel M., Kirkpatrick C. J., Weber A. (2006). *Actinomyces* in infected osteoradionecrosis—underestimated?. *Human Pathology*.

